# Opposing needling in the treatment of knee osteoarthritis: an improved study protocol for a randomized controlled trial

**DOI:** 10.3389/fmed.2025.1698983

**Published:** 2025-11-25

**Authors:** Huiying Li, Siyu Qian, Yanqin Liu, Yan Guo, Xiaoqin Chen, Yao Hu, Qiyue Fu, Qing Li, Yuting Xiang, Ying Liu, Xi Wu, Tingting Luo

**Affiliations:** 1 School of Acupuncture and Tuina, Chengdu University of Traditional Chinese Medicine, Chengdu, Sichuan, China; 2 Department of Rehabilitation, Chengdu Pidu District Traditional Chinese Medicine Hospital, Chengdu, Sichuan, China; 3 Rehabilitation Department, The Thirteenth People's Hospital of Chongqing, Chongqing, China

**Keywords:** knee, osteoarthritis, opposing needling, electroacupuncture, randomized controlled trial, evidence-based Chinese medicine

## Abstract

**Background:**

Knee osteoarthritis (KOA) is a common degenerative joint disease that typically affects individuals over 40 years of age and is characterized by knee pain, stiffness, and functional impairment. Current treatment options, including basic care, pharmacologic therapy, and surgery, often provide limited pain relief or are associated with adverse effects, high costs, and surgical risks. Acupuncture has been conditionally recommended by international guidelines for KOA management and has shown beneficial effects in alleviating pain and improving function. However, most studies have investigated single modalities such as manual acupuncture or electroacupuncture, leaving opportunities to explore strategies that may further enhance therapeutic outcomes. Opposing needling, a classical technique involving contralateral point selection, has demonstrated superior analgesic effects in various chronic pain conditions and is increasingly applied in KOA.

**Methods:**

This two-center randomized controlled trial will enroll 108 eligible patients with KOA, who will be randomly assigned in a 1:1:1 ratio to opposing needling plus electroacupuncture, sham opposing needling plus electroacupuncture, or electroacupuncture alone. The 12-week study comprises an 8-week intervention period and a 4-week follow-up, with treatments administered three times per week. The primary outcome will be the Western Ontario and McMaster Universities Osteoarthritis Index (WOMAC), while secondary outcomes will include WOMAC subscales, Visual Analog Scale, Numeric Rating Scale, Global Pain Scale, Lequesne Index, Knee Injury and Osteoarthritis Outcome Score, and Short Form-36 Health Survey.

**Discussion:**

The findings are expected to provide robust evidence regarding the efficacy and safety of opposing needling as an adjunctive therapy for KOA and to inform optimized treatment strategies.

**Clinical trial registration:**

itmctr.ccebtcm.org.cn, ITMCTR2025001291.

## 1 Introduction

Knee osteoarthritis (KOA) is a common chronic degenerative joint disorder predominantly affecting individuals over 40 years of age, characterized by progressive knee pain, stiffness, and functional impairment (1). It can lead to joint deformity and disability (2), imposing substantial burdens on patients, families, and healthcare systems. Current treatments, including basic care, pharmacologic therapy, and surgery (3), have limitations: basic interventions improve general condition but rarely relieve pain (3, 4); pharmacologic management may reduce inflammation and slow cartilage degeneration but carries risks such as nephrotoxicity and gastrointestinal or cardiovascular complications (5–8); surgical management is effective but costly and associated with postoperative pain, limited mobility, and prosthetic lifespan concerns (9). Thus, identifying effective, low-cost, and low-risk therapeutic strategies is essential for delaying disease progression and improving quality of life.

Acupuncture has demonstrated analgesic efficacy in chronic pain conditions and is conditionally recommended by clinical guidelines for KOA management, including the 2019 American College of Rheumatology (ACR) and Arthritis Foundation guideline (10). A systematic review indicated that acupuncture has both short-term and long-term efficacy in the treatment of KOA (11). Moreover, acupuncture has been recognized by the National Institutes of Health (NIH) as an important complementary and alternative therapy for KOA management (12). Electroacupuncture—a form of acupuncture combined with electrical stimulation—has been shown to promote cartilage repair and provide faster and superior pain relief compared with manual acupuncture (13–15). Multiple preclinical studies have shown that electroacupuncture can exert analgesic effects through various mechanisms (16), including the promotion of endogenous analgesic substance release (17), inhibition of pronociceptive mediator production (18), modulation of the MAPK signaling pathway (19), and suppression of pain sensitization (20).

Opposing needling, a classical technique involving contralateral acupoints with deep insertion along meridians, is primarily indicated for pain (21, 22) and has documented efficacy in various chronic pain disorders (23–27). From the perspective of traditional Chinese medicine, opposing needling emphasizes the concept of bodily integrity. When the flow of qi and blood along the meridians is obstructed and the left–right balance is disrupted, the meridian function on the affected side is weakened while the healthy side remains normal. Therefore, acupuncture at the corresponding points on the healthy side can stimulate the flow of qi, promote circulation of blood and qi, eliminate pathogenic factors, and restore the function of the affected meridians, achieving a dynamic balance of “yin and yang” between both sides of the body. From the perspective of modern medicine, studies have demonstrated that opposing needling can effectively relieve pain through protective inhibition of the cerebral cortex, reflex regulation via the nervous and humoral systems, and interruption of local nociceptive stimuli. The signals generated by acupuncture not only modulate pain transmission through spinal segmental connections but also promote the release of multiple endogenous analgesic substances, thereby increasing pain thresholds and activating descending inhibitory signals from higher centers. These combined effects contribute to the improvement of local pain and provide comprehensive pain management (28).

Previous studies suggest that opposing needling may enhance therapeutic outcomes in KOA, either alone or combined with other treatments (29–32). This indicates that opposing needling not only exerts an independent analgesic effect but may also play an adjunctive role. Therefore, we propose a reasonable hypothesis that combining opposing needling with electroacupuncture may produce additive or synergistic analgesic effects based on the analgesic action of electroacupuncture.

Given the independent and potential synergistic effects of opposing needling, we designed a sham-controlled, two-center randomized trial to evaluate the efficacy and safety of opposing needling as an adjunct to electroacupuncture in KOA patients. The study implements improved control procedures, comprehensive outcome measures, and refined group allocation to provide robust evidence and novel insights for clinical management.

## 2 Methods

### 2.1 Trial design

**Figure 1:**
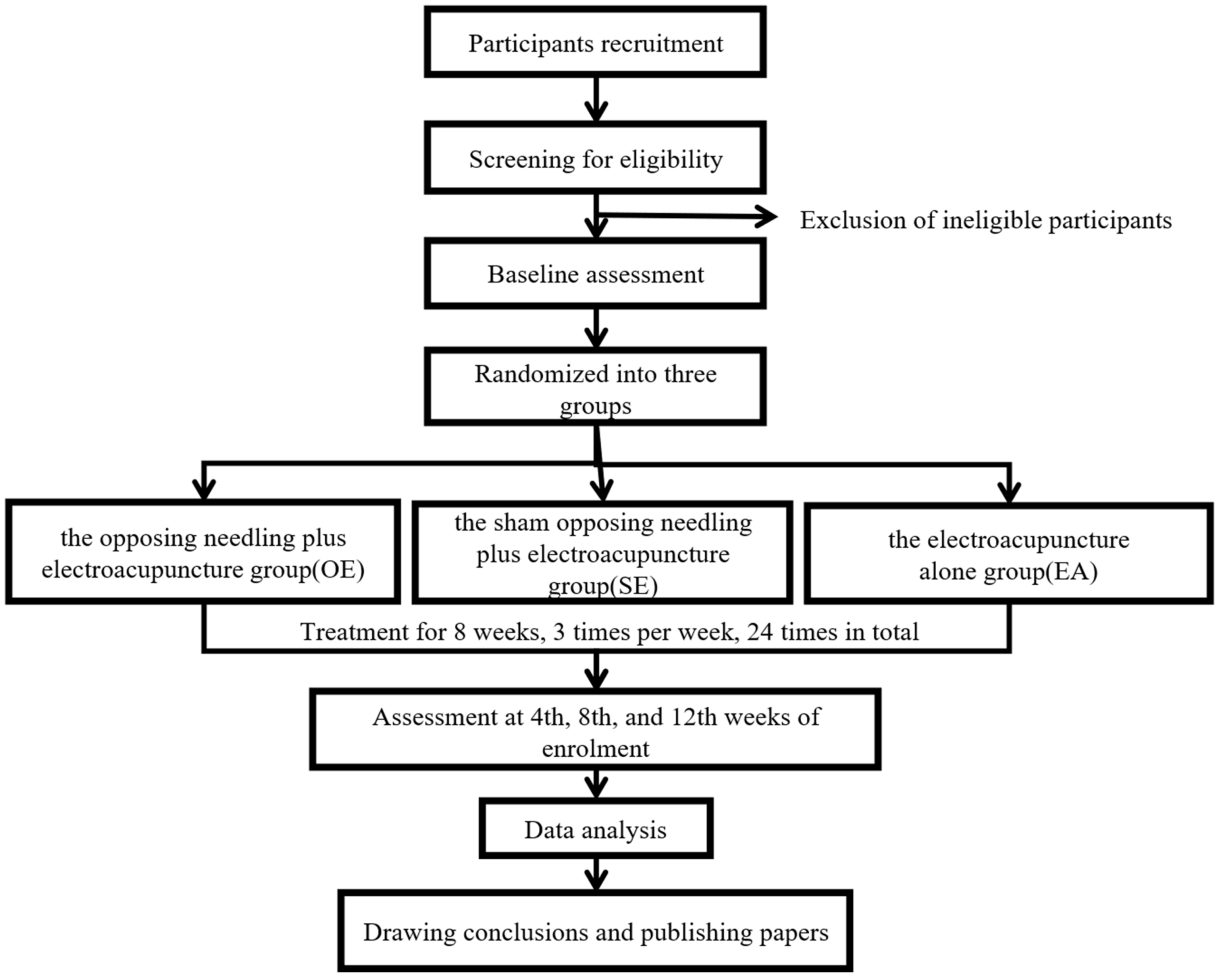
Flow chart for clinical trial procedure.

This is a three-armed RCT conducted at the Rehabilitation Department of the Thirteenth People’s Hospital of Chongqing and the Rehabilitation Department of Chengdu Pidu District Traditional Chinese Medicine Hospital. The total duration of the trial will be 12 weeks, comprising an 8-week intervention period followed by a 4-week follow-up period. A flow chart of the clinical trial procedure is presented in Figure 1, and the schedule of outcome measurements is detailed in Supplementary File 1 and Table 1.

**Table 1:** Schedule of outcome measurements.

Study period	Baseline	Treatment	End of treatment	Follow-up
Assessment point/time	0 weeks/1st	4 weeks/2nd	8 weeks/3rd	12 weeks/4th
Diagnosis and treatment				
Current/past medical history	×			
Physical examination	×			
Knee X-ray	×			
Informed consent	×			
Specialist assessment				
WOMAC	×	×	×	×
VAS	×	×	×	
NRS	×	×	×	×
GPS	×	×	×	
Lequesne	×	×	×	
KOOS	×	×	×	
SF-36	×	×	×	×
Trial assessment				
Adverse event assessment			×	
compliance assessment			×	
Clinical trial completion			×	
security assessment			×	
Post-completion work on test cases				
The person in charge reviews the case	Observations are reviewed and signed by the person in charge within 1 week of the end of the observation and entered into the electronic database.			
Review of medical cases by the Ombudsman	Regular monitoring and auditing by subject matter ombudsmen			

### 2.2 Participants

#### 2.2.1 Recruitment

Participants will be recruited from the outpatient clinic of the Rehabilitation Department at Thirteenth People’s Hospital of Chongqing and the Rehabilitation Department at Chengdu Pidu District Traditional Chinese Medicine Hospital, as well as through offline recruitment posters and online social media platforms.

#### 2.2.2 Diagnostic criteria for KOA

Only patients who meet the diagnostic criteria for KOA (based on the 1995 American College of Rheumatology classification criteria) will be eligible for inclusion in this study. Patients must have experienced recurrent knee pain within the past month and meet at least two of the following four criteria:

– Age ≥40 years;
– Morning stiffness ≤30 min;
– Crepitus on motion;
– Radiographic evidence on standing or weight-bearing X-rays demonstrating joint space narrowing, subchondral sclerosis and/or cystic changes, and osteophyte formation at the joint margins.

#### 2.2.3 Inclusion criteria

Only patients who meet all of the following five criteria will be eligible for inclusion in this study:

– Diagnosed with KOA according to the specified diagnostic criteria;
– Age ≥40 years, with knee pain persisting for at least 6 months;
– Radiographic evidence of Kellgren–Lawrence Grade II or III KOA (Grade II: definite osteophyte formation with possible joint space narrowing; Grade III: moderate osteophyte formation with definite joint space narrowing and subchondral sclerosis);
– NRS pain score ≥4 within the past week (scale from 0 to 10, with higher scores indicating more severe pain);
– Willingness to participate in the study and provide written informed consent.

#### 2.2.4 Exclusion criteria

Participants will be excluded if they meet any of the following criteria:

– Significant inflammatory response in the affected knee, such as visible swelling or moderate to large joint effusion;
– Knee pain caused by other diseases, including but not limited to rheumatoid arthritis or infectious arthritis;
– History of knee joint replacement surgery, or scheduled for joint replacement during the study period;
– Arthroscopic surgery within the past 12 months or intra-articular injection within the past 6 months;
– Receipt of acupuncture treatments within the past 3 months;
– Presence of severe acute or chronic organic diseases or psychiatric disorders, such as heart failure, severe arrhythmias, angina pectoris, renal failure, hepatic failure, malignancies, chronic obstructive pulmonary disease (COPD), dementia, or schizophrenia;
– Coagulation disorders or presence of implanted metal devices (e.g., cardiac pacemakers, deep brain stimulators);
– Needle phobia or known allergy to metal;
– Pregnant or breastfeeding women;
– Participation in another clinical trial within the past 3 months or currently enrolled in another trial.

#### 2.2.5 Withdrawal/dropout criteria

– Participants who are unable to tolerate acupuncture or fail to adhere to the designated study protocol;
– Participants who develop severe joint inflammation during the study, characterized by significant swelling or moderate to large joint effusion;
– Participants who experience other medical conditions requiring hospitalization, such as acute myocardial infarction or ischemic stroke.

#### 2.2.6 Management of withdrawal/dropout cases

– In the event of a participant dropout, the primary investigator should make efforts to contact the participant using methods such as home visits, phone calls, or letters to inquire about the reason for withdrawal. The date of the last treatment session should be recorded, and all feasible assessments should be completed.
– For participants who withdraw due to adverse reactions or treatment failure, the primary investigator should provide appropriate treatment based on the participant’s specific condition.
– Once a participant has been assigned a randomization number, they will be considered part of the study, regardless of subsequent changes in diagnosis or treatment completion.
– All withdrawal/dropout cases will undergo intention-to-treat analysis after the study is completed.

### 2.3 Sample size

The sample size was estimated based on preliminary data (33, 34). The mean and standard deviation (SD) of the change in total WOMAC score after 8 weeks of treatment were assumed to be −32.3 ± 1.15 in the opposing needling plus electroacupuncture group, −22.42 ± 2.32 in the sham opposing needling plus electroacupuncture group, and −8.87 ± 0.81 in the conventional electroacupuncture group. The test power (1 − *β*) was set at 0.9, and the significance level (*α*) was set at 0.05. Based on these parameters, 30 participants were required per group. Considering a 20% dropout rate, the final sample size was determined to be 36 participants per group, resulting in a total of 108 participants.

### 2.4 Randomization

Eligible participants will be randomly assigned in a 1:1:1 ratio to the opposing needling plus electroacupuncture (OE) group, sham opposing needling plus electroacupuncture (SE) group, or electroacupuncture alone (EA) group, according to the order of enrollment. The randomization sequence will be generated by an independent third party not involved in this study using SAS 9.4, with a stratified randomization method; the stratification factor is study center, and the block size is 6. Group allocation will be concealed in sequentially numbered, opaque, sealed envelopes. The envelopes will be opened by the acupuncturist immediately before the first treatment to determine group assignment.

### 2.5 Blinding

Participants, outcome assessors, and statisticians will be blinded to group allocation throughout the study. To maintain blinding, the three groups will be referred to as the OE group, SE group, and EA group, respectively. Due to the particularity of acupuncture, acupuncturists will not be blinded. Acupuncture treatments, outcome assessments, and statistical analyses will be conducted independently by different members of the research team.

### 2.6 Study groups and interventions

In this trial, acupuncture treatments will be performed by trained and certified acupuncturists with at least 500 h of clinical experience. Only those who have completed the standardized clinical operation training for this study and passed the qualification assessment are eligible to perform acupuncture. The training is conducted in strict accordance with the study’s Standard Operating Procedure (SOP) and includes communication skills, detailed acupuncture operation specifications (needle insertion depth, manipulation techniques, rotation angle and frequency, and Deqi elicitation), and the use of Park sham needles. Upon completion of the training, acupuncturists must pass the assessment and sign a “Declaration of SOP Acknowledgment and Compliance.” During the trial, monitoring staff will perform regular checks to ensure that acupuncture procedures are strictly standardized.

#### 2.6.1 Opposing needling plus electroacupuncture (OE)

**Figure 2:**
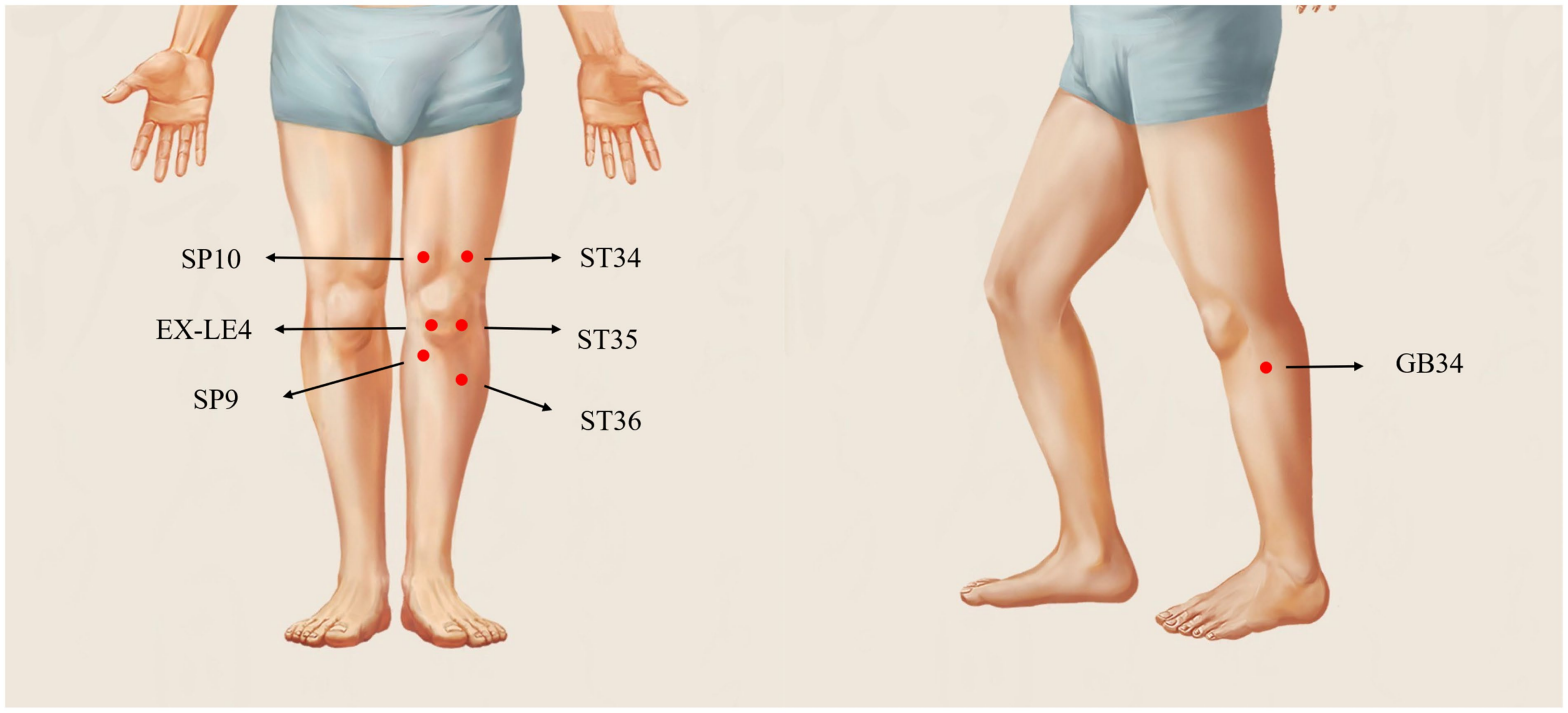
Acupuncture point diagram. Screenshot from the mobile application ‘Zhiyuan Meridian and Acupoint’ (知源经络穴位) (https://apps.apple.com/app/id1554030035), with local knee acupoints labeled by the authors. Copyright permission for academic use has been obtained.

Acupuncture will be administered bilaterally, including both the side with more significant pain and the contralateral side, which presents with mild or no pain. The selected acupoints include, Zusanli (ST36, bilateral), Yanglingquan (GB34, bilateral), Xuehai (SP10, bilateral), Liangqiu (ST34, bilateral), Dubi (ST35, more affected side), Neixiyian (EX-LE4, more affected side), Yinlingquan (SP9, more affected side), and Ashi points (more affected side). Acupoint locations will be determined according to the National Standard of the People’s Republic of China, *Nomenclature and Location of Acupuncture Points* (GB/T 12346–2021). Figure 2 illustrates the anatomical locations of these acupoints.

This trial will use Chinese HWATO stainless steel filiform needles (0.30 mm × 25 mm and 0.30 mm × 40 mm) and the Chinese HWATO SDZ-III Low-Frequency (1–100 Hz) Pulse Electronic Acupuncture Stimulator Machine, with 6 channels, and adjustable output power (0.3–10 V at 250 ohms).

Prior to the procedure, the acupuncturists will wash their hands with antiseptic hand sanitizer and disinfect the skin at each acupoint site using a 75% alcohol swab in a circular motion from the center outward. Needle insertion depth will be determined based on the specific anatomical characteristics of each acupoint. The twisting technique will be used to elicit Deqi sensation, characterized by soreness, numbness, heaviness, distension, warmth, coolness, or the transmission of needling sensation, while the acupuncturists perceive resistance or tension beneath the needle.

Once Deqi sensation is achieved, a twirling-rotating method with even manipulation will be applied using a twisting angle of approximately 180° at a frequency of 120–160 times per minute. Electroacupuncture will be applied to the more affected knee for 30 min. Two pairs of output wires will be connected: one pair from Liangqiu (ST34, anode) to Yanglingquan (GB34, cathode) on the lateral side, and another from Yinlingquan (SP9, anode) to Xuehai (SP10, cathode) on the medial side. A dense-disperse wave of 2/100 Hz will be selected, and the electrical current will be gradually increased until the patient experiences a mild, rhythmic muscle twitching around the acupoints without discomfort.

After 30 min of stimulation, the acupuncturists will press a dry cotton ball around the needle site with one hand and simultaneously rotate and withdraw the needle slowly with the other hand. Gentle pressure will be applied to the insertion site to prevent bleeding.

#### 2.6.2 Sham opposing needling plus electroacupuncture (SE)

**Figure 3:**
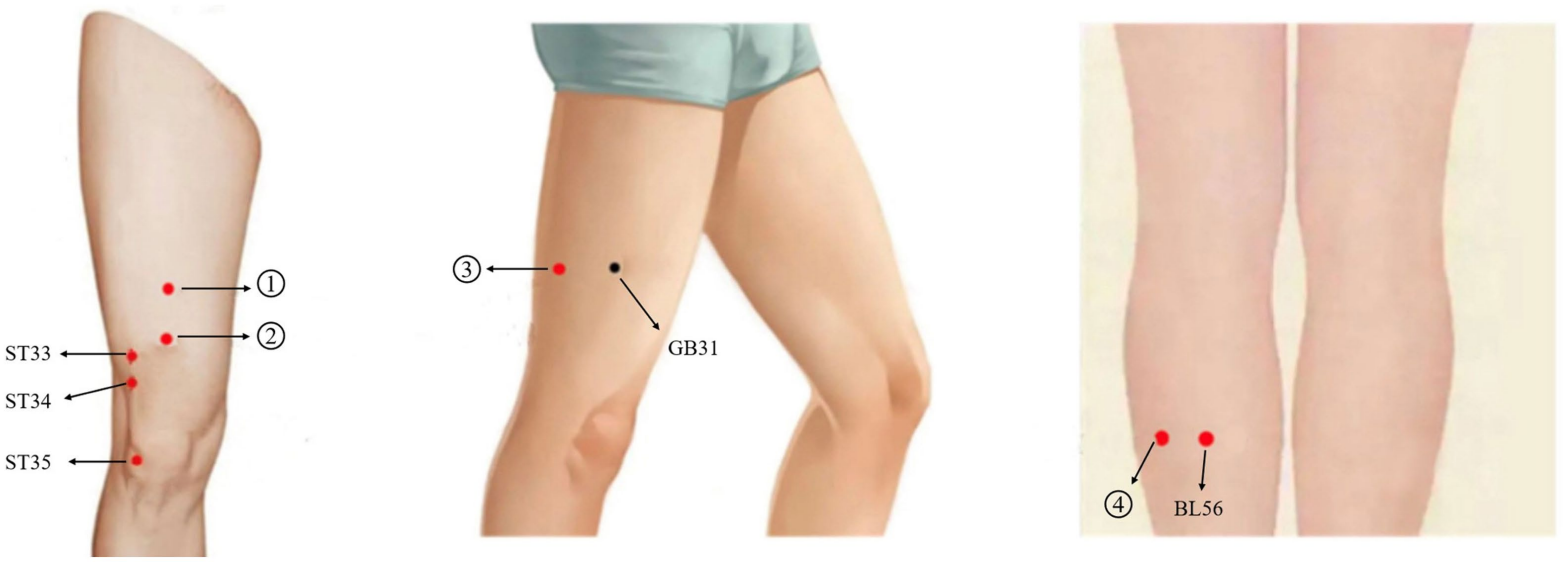
Diagram of non-meridian and non-acupoint locations for the SE group. Screenshot from the mobile application ‘Zhiyuan Meridian and Acupoint’ (知源经络穴位) (https://apps.apple.com/app/id1554030035), with local knee acupoints labeled by the authors. Copyright permission for academic use has been obtained.

Both knees received acupuncture treatments. The acupuncture protocol on the more affected side was identical to that used in the OE group, while four non-acupoint, non-meridian sites on the contralateral calf—distant from the knee and with mild or no pain—were selected for sham opposing needling. The anatomical locations of these sites are illustrated in Figure 3 and detailed in Supplementary File 1 and Table 2.

**Table 2:** Detailed description of non- meridian and non-acupoint locations.

Non-meridian and non-acupoint	Acupoint localization
1	6 cun superior to the patella, approximately 1 cun (roughly 2 cm) between the Spleen and Stomach meridians
2	4 cun superior to the patella, between the Spleen and Stomach meridians
3	2 cun posterior to Fengshi (GB31), avoiding the Bladder meridian
4	1 cun medial to Chengjin (BL56)

**Figure 4:**
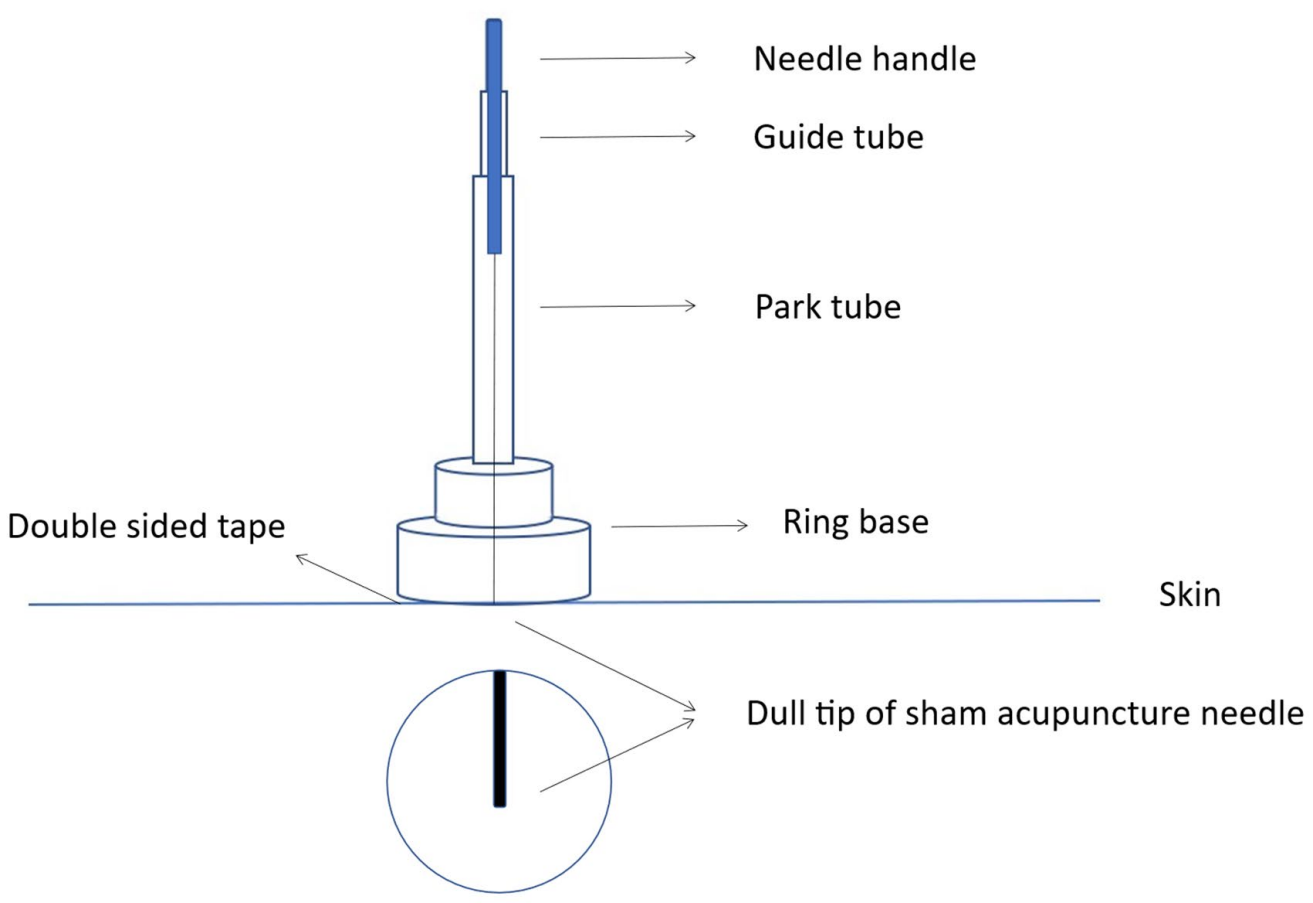
Structural principle of the Park sham device.

Sham opposing needling will be performed using the Park sham device (35), which features a blunt, retractable needle tip designed to mimic the sensation of skin penetration without actual insertion. Each needle is supported by a circular adhesive base affixed to the skin surface. The structural principle of the Park sham device is illustrated in Figure 4.

All four non-acupoint sites will undergo perpendicular sham opposing needling. Because of the blunt needle tip, the skin will not be penetrated, and the needles will be supported by the circular bases. To minimize bias from psychological effects, a standardized twirling–rotating manipulation, including amplitude and frequency, will be performed to match the procedure used in the OE group.

After the 30-min intervention, the sham device will be removed. The acupuncturists will press a dry cotton ball over each site while simulating needle withdrawal using the same technique as in the OE group to ensure consistency in the treatment procedure.

#### 2.6.3 Electroacupuncture alone

The more affected side will receive acupuncture treatment following the same needling procedures, acupoint selection, and electroacupuncture protocol as the more affected side in the OE group.

#### 2.6.4 Rescue therapy

During the treatment period, if the participant experiences a significant exacerbation of pain that becomes intolerable, a rescue dose of celecoxib (200 mg) will be permitted. The acupuncturists will record the use of any rescue therapy in detail. Aside from this provision, the use of any additional analgesics is strictly prohibited throughout the study period. Participants will also be prohibited from receiving any other treatments related to KOA, including joint injections of any kind, moxibustion, cupping, tuina massage, or similar interventions.

#### 2.6.5 Basic treatment

1. All enrolled participants will receive basic health education, with the content based on the Consensus of Four-Stepladder Program of Knee Osteoarthritis (2019) issued by the Chinese Orthopedic Association.
  The specific educational components are as follows:
  (1) Providing patients with information about KOA to enhance their understanding of the disease;
  (2) Instructing patients on weight management through dietary modifications and functional exercise, advising them to avoid unnecessary weight-bearing activities (e.g., carrying heavy objects, climbing stair), refraining from excessive physical exertion to prevent joint injury, and maintaining warmth to protect the joints;
  (3) Encouraging patients to engage in appropriate muscle-strengthening exercises for the joints, such as aerobic activities, Tai Chi, and Baduanjin.

#### 2.6.6 Treatment schedule

Each treatment session will last for 30 min. Participants will receive treatments three times per week, over a period of 8 consecutive weeks, for a total of 24 sessions.

#### 2.6.7 Baseline information

On day 0 of the trial, general clinical data will be collected for all participants, including disease duration (months), affected knee(s), radiographic grading (X-ray), prior treatments (medications, pharmacologic therapy, electroacupuncture or opposing needling, etc.), and concomitant systemic diseases. These baseline data will be used to describe participant characteristics and to account for potential confounding factors in the statistical analysis, providing a reference for the assessment of primary, secondary, and other observational outcomes.

### 2.7 Outcome measurements

All outcome measures will be assessed at baseline, at the end of the 4th week, and at the end of the 8th week. Additionally, WOMAC, NRS, and SF-36 will be reassessed at the end of the 12th week to evaluate changes in intervention effects across the OE, CE, and EA groups.

#### 2.7.1 Primary outcome

##### 2.7.1.1 The Chinese version of the Western Ontario and McMaster universities osteoarthritis index (WOMAC) score for the more affected knee

During the trial, the WOMAC score of the more affected knee will be assessed at baseline and at weeks 4, 8, and 12. The primary analysis will focus on the change from baseline to week 8 to evaluate the treatment effect, while scores at other time points will be used to assess the trend and maintenance of efficacy.

#### 2.7.2 Secondary outcomes

##### 2.7.2.1 WOMAC pain subscale score

During the trial, the WOMAC pain subscale score of the more affected knee will be assessed at baseline and at weeks 4, 8, and 12. The primary analysis will focus on the change from baseline to week 8 to evaluate the treatment effect, while scores at other time points will be used to assess the trend and maintenance of efficacy.

##### 2.7.2.2 WOMAC stiffness subscale score

During the trial, the WOMAC stiffness subscale score of the more affected knee will be assessed at baseline and at weeks 4, 8, and 12. The primary analysis will focus on the change from baseline to week 8 to evaluate the treatment effect, while scores at other time points will be used to assess the trend and maintenance of efficacy.

##### 2.7.2.3 WOMAC function subscale score

During the trial, the WOMAC physical function subscale score of the more affected knee will be assessed at baseline and at weeks 4, 8, and 12. The primary analysis will focus on the change from baseline to week 8 to evaluate the treatment effect, while scores at other time points will be used to assess the trend and maintenance of efficacy.

##### 2.7.2.4 Visual analog scale

During the trial, the VAS score of the more affected knee will be assessed at baseline and at weeks 4, 8, and 12. The primary analysis will focus on the change from baseline to week 8 to evaluate the treatment effect, while scores at other time points will be used to assess the trend and maintenance of efficacy.

##### 2.7.2.5 Numerical rating scale

During the trial, the NRS score of the more affected knee will be assessed at baseline and at weeks 4, 8, and 12. The primary analysis will focus on the change from baseline to week 8 to evaluate the treatment effect, while scores at other time points will be used to assess the trend and maintenance of efficacy.

##### 2.7.2.6 Global pain scale

During the trial, the GPS score of the more affected knee will be assessed at baseline and at weeks 4, 8, and 12. The primary analysis will focus on the change from baseline to week 8 to evaluate the treatment effect, while scores at other time points will be used to assess the trend and maintenance of efficacy.

##### 2.7.2.7 Lequesne functional index (Lequesne index)

During the trial, the Lequesne index score of the more affected knee will be assessed at baseline and at weeks 4, 8, and 12. The primary analysis will focus on the change from baseline to week 8 to evaluate the treatment effect, while scores at other time points will be used to assess the trend and maintenance of efficacy.

##### 2.7.2.8 Knee injury and osteoarthritis outcome score

During the trial, the KOOS of the more affected knee will be assessed at baseline and at weeks 4, 8, and 12. The primary analysis will focus on the change from baseline to week 8 to evaluate the treatment effect, while scores at other time points will be used to assess the trend and maintenance of efficacy.

##### 2.7.2.9 Short form 36 health survey (SF-36)

The SF-36 subscales of physical functioning, role physical, bodily pain, general health, vitality, social functioning, role emotional, and mental health, as well as the total score, of the more affected knee will be assessed at baseline and at weeks 4, 8, and 12. The primary analysis will focus on the change from baseline to week 8 to evaluate the treatment effect, while scores at other time points will be used to assess the trend and maintenance of efficacy.

### 2.8 Adverse events

All trial investigators, responsible units, clinical monitors, and participants must record and promptly report any AEs to the project leader and study team, regardless of their relationship to the study intervention. AEs should be documented in the source documents and case report forms (CRFs), including a description of the event, onset time, duration, severity, frequency, interventions administered, outcome, and the investigator’s assessment of causality. Common AEs associated with acupuncture and electroacupuncture include fainting, needle retention, needle breakage, local pain, bleeding, or subcutaneous bruising, which are generally mild to moderate. Investigators should provide appropriate symptomatic management according to the participant’s condition and continue follow-up until the participant’s condition stabilizes. Relevant clinical data should be properly preserved to support AE evaluation and reporting.

### 2.9 Serious adverse events

SAEs include events that lead to hospitalization, prolongation of hospitalization, persistent or significant functional impairment, are life-threatening, result in death, or are considered by medical judgment to require intervention to prevent the occurrence of the above serious outcomes. In the event of an SAE, the clinical physician must immediately (within 2 h) report it to the project leader and the study team. The study team shall report the SAE to the ethics committee and the sponsor within 24 h. The SAE must be recorded in detail in the CRF, including the time of occurrence, severity, outcome, causal relationship, reporting status, and management process. Appropriate management should be implemented according to the participant’s condition, and consultation with relevant departments or hospitalization may be arranged if necessary. If emergency unblinding is required to confirm the treatment received, the participant will be withdrawn from the trial and recorded as a dropout. The study team will continue to follow up until the participant’s condition stabilizes. All costs arising from the management of SAEs and AEs will be covered by the study-designated funding.

### 2.10 Attendance and drop-out

Participants will be informed that they have the right to withdraw from the clinical trial at any time without obligation. The reasons for withdrawal will be tracked and recorded. Participants are not required to provide a reason for withdrawal; however, if the withdrawal is related to an AE, the investigator will take necessary measures based on the participant’s condition until it stabilizes. In the case of abnormal laboratory results, follow-up will continue until normalization is achieved. The method of follow-up, including hospitalization, outpatient visits, home visits, or telephone follow-up, will be determined based on the severity of the AE. Participants who receive other treatments or enroll in other clinical trials during the study will be considered as withdrawn from the trial.

### 2.11 Data management

A dedicated database system for this trial will be developed using EpiData version 3.1 (EpiData Association, Denmark), based on the items in the CRFs. Logical checks and validation constraints will be incorporated during data entry. A trial run will be conducted to ensure the functionality and integrity of the system. Once the CRFs have been reviewed and signed, they will be submitted to the data manager, who will verify the data, including inclusion and exclusion criteria, dropout status, and missing values. If any inconsistencies or ambiguities are identified, a “data issue form” will be completed and returned to the clinical monitor for clarification. The investigator will provide written explanations, sign the form, and return it to the data manager. All data issue forms will be securely archived. Data entry will be performed using a double data entry method, in which two trained personnel independently enter the data. Discrepancies between entries will be resolved by comparing them against the original source documents and making necessary corrections.

All personnel involved in data management will receive training on the project background, CRF structure and coding, and data entry procedures. Data verification will be conducted using EpiData 3.1 by cross-checking each entry against the original CRF. To ensure data accuracy, random verification of 10 cases will be performed, comparing database entries with the corresponding CRFs to confirm consistency.

### 2.12 Statistical analysis

Demographic Data Analysis: To assess the comparability between the three groups, demographic data and other baseline characteristics were compared. Continuous variables, such as age and Body Mass Index (BMI), were analyzed using independent-samples *t*-tests, provided that the data met the normality assumption. If the data did not meet the normality assumption, non-parametric tests, such as the Kruskal-Wallis test, were used. Categorical variables, such as gender, were analyzed using χ^2^ tests, ensuring that the assumptions for the test (e.g., adequate sample size in each category) were satisfied.

Clinical Efficacy Analysis: A multivariate analysis of covariance (ANCOVA) was performed to assess efficacy outcomes, with age, BMI, and disease duration included as covariates. The primary and secondary outcome measures were clearly defined, and potential confounding factors were controlled to ensure the robustness of the results.

Safety Analysis: The incidence of AEs and SAEs at 8 weeks post-randomization was compared across the three groups using χ^2^ tests. All AEs and SAEs were documented and analyzed to assess the safety of the interventions in each group.

Handling of Missing Data: After data collection is completed, the proportion, distribution, and possible mechanisms of missing data [e.g., missing completely at random (MCAR) or missing at random (MAR)] will be examined. For outcomes with a low proportion of missing data (≤5%) (36) and no apparent systematic pattern, complete-case analysis will be performed. If the proportion of missing data is higher or systematic missingness is observed, multiple imputation will be applied under the assumption of missing at random to reduce bias and maximize data utilization. Sensitivity analyses will be conducted to assess the robustness of the results under different missing data handling approaches. All analyses will be performed using R software (R version 4.4.3).

## 3 Discussion

The present study is expected to provide robust evidence regarding the efficacy and safety of combining opposing needling with electroacupuncture for KOA. By testing the hypothesis that this combination may produce additive or synergistic analgesic effects, the trial aims to elucidate the therapeutic value of integrating these two classical acupuncture techniques. A rigorous three-arm parallel-group design will be employed, including an OE group, a SE group, and an EA group. On one hand, this design allows for the evaluation of the additive effect of opposing needling as well as the overall therapeutic benefit of the combined intervention; on the other hand, both the OE and SE groups receive bilateral needling, which helps reduce the risk of unblinding. In addition, the trial incorporates a comprehensive set of outcome measures, enabling a multidimensional assessment of the therapeutic effects of opposing needling plus electroacupuncture. Finally, the study emphasizes standardization and reproducibility of the intervention: all treatments will be administered by uniformly trained acupuncturists, and quality control and monitoring procedures are established to ensure intervention consistency and internal validity of the results.

Nevertheless, certain limitations remain. The follow-up period is limited to 12 weeks, allowing only preliminary evaluation of efficacy and precluding assessment of long-term effects in KOA; future studies should consider extending the follow-up duration. In addition, this is a two-center study with a relatively small sample size. Larger and multicenter RCTs will be required to further validate the generalizability and external applicability of the findings.

Overall, the results of this study are expected to strengthen the clinical evidence for acupuncture-based management of KOA, inform treatment optimization, and provide a foundation for future research on innovative needling techniques in chronic pain management.
