# Correction: Opposing needling in the treatment of knee osteoarthritis: an improved study protocol for a randomized controlled trial

**DOI:** 10.3389/fmed.2025.1762859

**Published:** 2026-01-05

**Authors:** Huiying Li, Siyu Qian, Yanqin Liu, Yan Guo, Xiaoqin Chen, Yao Hu, Qiyue Fu, Qing Li, Yuting Xiang, Ying Liu, Xi Wu, Tingting Luo

**Affiliations:** 1 School of Acupuncture and Tuina, Chengdu University of Traditional Chinese Medicine, Chengdu, Sichuan, China; 2 Department of Rehabilitation, Chengdu Pidu District Traditional Chinese Medicine Hospital, Chengdu, Sichuan, China; 3 Rehabilitation Department, The Thirteenth People's Hospital of Chongqing, Chongqing, China

**Keywords:** knee, osteoarthritis, opposing needling, electroacupuncture, randomized controlled trial, evidence-based Chinese medicine

The funder “Joint Fund of Chongqing Science and Technology Bureau and Health Commission, [Grant No. 2023QNXM063]” to Tingting Luo was erroneously omitted.

The correct Funding statement is:

“The author(s) declared that financial support was received for this work and/or its publication. This work was funded by Joint Fund of Chongqing Science and Technology Bureau and Health Commission, [Grant No. 2023QNXM063] to Tingting Luo.”

The original version of this article has been updated.

