# Supplementary material for: Opposing needling in the treatment of knee osteoarthritis: an improved study protocol for a randomized controlled trial
Source: Front Med (Lausanne). 2025 Nov 25;12:1698983. doi: 10.3389/fmed.2025.1698983 (PMC12687746; doi:10.3389/fmed.2025.1698983)
Supplement: Supplementary file 2 [file Supplementary_file_2.docx]

**Participant Information Sheet/Consent Form**

**Clinical Research Project Title:**

Efficacy and Safety Study of the opposing needling in the Treatment of Knee Osteoarthritis

**Protocol Number:** 2023QNXM063

**Project Undertaking Institution:**
Chongqing 13th People's Hospital; Chengdu Pidu District Traditional Chinese Medicine Hospital

**Collaborating Institutions:**

Chengdu University of Traditional Chinese Medicine

**Dear participant,**

Your doctor has diagnosed you with Knee Osteoarthritis (KOA). We sincerely invite you to participate in a research project titled "Efficacy and Safety Study of the opposing needling in the Treatment of Knee Osteoarthritis", funded by the Chongqing Municipal Science and Health Joint Youth Program in Traditional Chinese Medicine. The objective of this study is to evaluate the efficacy and safety of the opposing needling in treating KOA, providing scientific evidence for optimizing acupuncture treatment protocols for this condition.

This study is jointly conducted by Chongqing 13th People's Hospital, the School of Acupuncture and Tui Na at Chengdu University of Traditional Chinese Medicine, and the Chengdu Pidu District Traditional Chinese Medicine Hospital. A total of 108 participants will be enrolled, with 54 participants recruited at Chongqing 13th People's Hospital and 54 participants at the Chengdu Pidu District Traditional Chinese Medicine Hospital. The study will last for 36 months, and your participation will span 12 weeks.

Ethical approval for this study was obtained from the Medical Ethics Committees of Chongqing 13th People's Hospital and Chengdu Pidu District Traditional Chinese Medicine Hospital.

Before you decide whether to participate in this study, please read the following information carefully. This document will help you understand the purpose of the study, its procedures and duration, as well as the potential benefits, risks, and discomforts associated with participation. You are encouraged to discuss your participation with your family, friends, or a physician to assist you in making an informed decision. This document is part of the informed consent form for the clinical study.

**1 Background and objectives of the study**

KOA is a common chronic, progressive, and degenerative joint disease affecting adults aged 40 years and older. It is primarily characterized by articular cartilage degeneration, subchondral bone sclerosis, and osteophyte formation. The main clinical manifestations include joint pain, swelling, stiffness, and functional impairment. Recurrent pain and dysfunction of the knee joint significantly reduce patients’ quality of life and increase the risk of other chronic diseases. KOA has become a leading cause of disability among middle-aged and elderly individuals.

Acupuncture, as a traditional Chinese medicine (TCM) therapy, is an important non-pharmacological treatment for KOA, demonstrating both short-term and long-term efficacy in alleviating chronic pain and functional impairment associated with the condition. Opposing needling is a classical acupuncture method specifically described in the *Huangdi Neijing (Huangdi’s Canon of Medicine)* for treating chronic pain. It is characterized by contralateral acupoint selection and has been applied clinically in the treatment of KOA. However, its efficacy has not yet been substantiated by rigorous scientific evidence, limiting its widespread adoption.

Therefore, this study aims to compare the therapeutic effects of opposing needling, conventional acupuncture, and sham opposing needling in improving pain and functional impairment in KOA patients. By evaluating the efficacy and safety of opposing needling, this study seeks to provide scientific evidence for optimizing acupuncture treatment protocols for KOA.

**2 Eligibility criteria for participation**

– Age ≥40 years.

– Recurrent knee pain persisting for 6 months or longer.

– Knee pain not attributable to other diseases, such as rheumatoid arthritis or infectious arthritis.

– No significant inflammatory response in the knee joint, such as marked swelling or moderate to large joint effusion.

– Radiographic evidence of KOA on the pain-dominant side, as indicated by definite osteophyte formation, suspected joint space narrowing, or moderate osteophytes with confirmed joint space narrowing and sclerosis.

– No history of knee or hip joint replacement surgery and not planning to undergo joint replacement surgery.

– No history of arthroscopy within the past 12 months or intra-articular injection within the past 6 months.

– No acupuncture treatment in the past 3 months.

– No history of severe acute or chronic organic diseases or mental disorders, such as heart failure, severe arrhythmia, angina pectoris, renal failure, hepatic failure, chronic obstructive pulmonary disease (COPD), dementia, or schizophrenia.

– No coagulation disorders or metal implants (e.g., cardiac pacemakers, deep brain stimulators).

– No needle phobia or metal allergies.

– Not pregnant or breastfeeding.

– Not currently participating in or having participated in another clinical trial within the past 3 months.

**3 What will be required of you if you participate in the study?**

**(1) Before being included in the study, you will need to complete the following steps to determine your eligibility:**

The doctor will inquire about and record your medical history, perform a specialized examination, and assess joint pain, function, and quality of life.

**(2) If the screening results meet the inclusion criteria, the following steps will be taken:**

a. The doctor will use a computer-generated random number to assign you to one of three groups: the opposing needling plus electroacupuncture (OE) group, the sham opposing needling plus electroacupuncture (SE) group, and the electroacupuncture alone (EA) group. You will have a 1/3 chance of being assigned to any one of these groups, and neither you nor your doctor will know or be able to choose the treatment method in advance.

b. The study period will last for 12 weeks. You will receive treatment 3 times per week for 8 weeks, for a total of 24 sessions. A follow-up assessment will be conducted at week 12 to evaluate your response after the treatment.

c. Before the group assignment, you will undergo various evaluations, including the Western Ontario and McMaster Universities Osteoarthritis Index (WOMAC), Visual Analogue Scale (VAS), Numerical Rating Scale (NRS), Global Pain Scale (GPS), Lequesne Functional Index, Knee Injury and Osteoarthritis Outcome Score (KOOS), and the Short Form 36 Health Survey (SF-36).

d. After the 4th and 8th weeks of treatment, data will be collected again as described in (2)(c). Any adverse events (AEs) or serious adverse events (SAEs) during treatment will be evaluated.

**(3) Other Responsibilities:**

a. As a participant in this study, you will have certain responsibilities, such as attending hospital appointments for treatment and examinations, as well as participating in follow-up visits. Additionally, you must inform the doctor about any physical or psychological changes you experience during the trial, regardless of whether you think these changes are related to the study.

b. You must attend follow-up appointments as scheduled by the doctor. During the follow-up phase, the doctor may also contact you via phone, WeChat, or email to monitor your progress. Follow-up visits are crucial because the doctor will assess the effectiveness of the treatment and provide guidance on preventing or managing any related conditions.

c. During the study, you must refrain from using any other treatments for KOA, including pharmacological or non-pharmacological management. If you require other treatments, please contact your doctor in advance.

**4 Potential benefits of participating in the study**

You may benefit from participating in this study. Potential benefits include possible improvement in your KOA symptoms and receiving free physical examinations, imaging tests, scale assessments, and acupuncture treatments.

If you experience any adverse reactions or events during acupuncture treatment, please contact your doctor immediately for assistance. The doctor will provide appropriate treatment, and the treatment costs will be covered by the study. If any of these adverse reactions or events result in objective harm deemed to be related to the acupuncture treatment, compensation may be provided on a case-by-case basis.

It is important to note that acupuncture is not the only treatment method for KOA. You may ask your doctor about alternative treatments that could be available to you. Common alternative treatments include oral pain medications, intra-articular injections (such as corticosteroids and hyaluronic acid), and surgical managements (such as arthroscopic surgery and joint replacement). These treatments may help relieve pain and improve joint function to some extent. However, they also carry certain risks, such as gastrointestinal bleeding, serious cardiovascular events (including myocardial infarction and heart failure), cartilage loss or damage, deep vein thrombosis and pulmonary embolism, infection, poor wound healing, joint effusion, and synovitis. In addition, the medium- and long-term effectiveness of most of these treatments is often unsatisfactory.

**5 Potential adverse reactions, risks, discomforts, and inconveniences of participating in the study**

Acupuncture treatmens may cause some adverse reactions, including pain, needle fainting, bleeding, needle retention, needle breakage, or infection at the acupuncture site. The doctor will make every effort to prevent and treat any harm that may arise during the study. During acupuncture treatment, you may experience sensations such as aching, numbness, heaviness, or fullness, which are normal reactions to acupuncture treatment. However, some mild and less common adverse reactions may occur post-treatment, such as fainting due to your constitution or anxiety. This can usually be alleviated by stopping the acupuncture and resting. Minor bleeding may also occur at the acupuncture site but will typically stop after local pressure is applied. In the event of infection, needle retention, or needle breakage, the doctor will address the issue promptly, and you should cooperate actively with the treatment.

If you experience any discomfort during the study, notice changes in your condition, or encounter any unexpected situations—whether or not related to acupuncture—you should inform your doctor immediately. The doctor will assess the situation and provide appropriate medical treatment.

You will need to attend the hospital regularly for treatment and undergo specialized evaluations, which may cause some inconvenience or discomfort.

If you have any questions or concerns regarding the study, you may contact the project leader, Tingting Luo, at 18782007605.

**6 Study costs**

During the study, all physical examinations, imaging tests, health guidance and acupuncture treatments will be provided free of charge.

The doctor will make every effort to prevent and treat any potential harm caused by this study. If AEs occur during the clinical trial, a medical expert committee will assess whether they are related to the acupuncture treatment. The study sponsor will cover the treatment costs and provide appropriate financial compensation for any harm directly related to the trial procedures.

However, any treatment and examinations required for other coexisting medical conditions that you may have will not be covered by this study.

**7 Is your personal information confidential?**

Your medical records (including CRF forms, test reports, etc.) will be securely stored at the research institution. The clinical trial organizers, the ethics committee, and the clinical trial research center will be permitted to review your medical records as necessary.

Any public reports related to this study’s findings will not disclose your personal identity. We will make every effort, within the limits of the law, to protect the privacy of your personal medical information.

Your data will only be used for this study and will not be used for any other research purposes.

**8 How can you obtain more information?**

You may ask any questions regarding this study at any time and receive appropriate answers. If any important new information arises during the study that may affect your willingness to continue participating, your doctor will inform you promptly.

**9 Voluntary participation and withdrawal**

Your participation in this study is entirely voluntary. You have the right to refuse to participate or withdraw at any time during the study. This decision will not affect your relationship with your doctor, your medical care, or any other benefits to which you are entitled. For your best interests, the doctor or researcher may also decide to discontinue your participation in the study if necessary.

If you choose to withdraw for any reason, you may be asked about your acupuncture treatment or medication usage. If deemed necessary by the doctor, you may also be asked to undergo a physical examination and specialized assessment.

**10 What should you do next?**

The decision to participate in this study is entirely up to you (and your family). Before making your decision, please feel free to ask your doctor any questions you may have about the study.

Thank you for taking the time to read this information. If you decide to participate, please inform your doctor, who will make all the necessary arrangements for your participation.

Please keep this document for your records.

**Informed Consent Form – Signature Page**

**Clinical Research Project Title:**

Efficacy and Safety Study of the opposing needling in the Treatment of Knee Osteoarthritis

**Protocol Number:** 2023QNXM063

**Project Undertaking Institution:**
Chongqing 13th People's Hospital; Chengdu Pidu District Traditional Chinese Medicine Hospital

**Collaborating Institutions:**

Chengdu University of Traditional Chinese Medicine

**Statement of Consent**

I have read the above introduction to this study and have had the opportunity to discuss it with my doctor and ask questions. All my questions have been answered satisfactorily.

I understand the potential risks and benefits of participating in this study. I acknowledge that my participation is voluntary, and I have had sufficient time to consider my decision. I also understand that:

- I can consult my doctor for more information at any time.
- I can withdraw from this study at any time without facing discrimination or retaliation, and my medical care and rights will not be affected.

I also acknowledge that if I withdraw from the study, particularly due to reasons related to acupuncture treatment, informing my doctor of any changes in my condition and completing the necessary physical examinations and specialist assessments will be beneficial to the study.

If my condition changes and requires alternative treatment, I will consult my doctor beforehand or inform them truthfully afterward.

I agree to allow the Ethics Committee or the study sponsor’s representatives to review my research data.

I will receive a signed and dated copy of this informed consent form.

Finally, I agree to participate in this study and commit to following medical instructions to the best of my ability.

Patient Signature

Contact Number Date

I confirm that I have explained the details of this study to the patient, including their rights, potential benefits, and risks, and have provided them with a signed copy of the informed consent form.

Doctor's Signature

Doctor's Work Phone Number Date
